# Enhanced autocrine FGF19/FGFR4 signaling drives the progression of lung squamous cell carcinoma, which responds to mTOR inhibitor AZD2104

**DOI:** 10.1038/s41388-020-1227-2

**Published:** 2020-02-28

**Authors:** Fan Li, Ziming Li, Qing Han, Yirui Cheng, Wenxiang Ji, Ying Yang, Shun Lu, Weiliang Xia

**Affiliations:** 1 State Key Laboratory of Oncogenes and Related Genes, Renji-Med X Clinical Stem Cell Research Center, Ren Ji Hospital, School of Medicine and School of Biomedical Engineering, Shanghai Jiao Tong University, Shanghai, China; 2 Shanghai Lung Cancer Center, Shanghai Chest Hospital, Shanghai Jiao Tong University, Shanghai, China

**Keywords:** Non-small-cell lung cancer, Oncogenes

## Abstract

Lung cancer occurrence and associated mortality ranks top in all countries. Despite the rapid development of targeted and immune therapies, many patients experience relapse within a few years. It is urgent to uncover the mechanisms that drive lung cancer progression and identify novel molecular targets. Our group has previously identified FGF19 as a prognostic marker and potential driver gene of lung squamous cell carcinomas (LSQ) in Chinese smoking patients. However, the underlying mechanism of how FGF19 promotes the progression of LSQ remains unclear. In this study, we characterized and confirmed that FGF19 serves as an oncogenic driver in LSQ development and progression, and reported that the amplification and high expression of FGF19 in LSQ was significantly associated with poor overall and progression-free survival. A higher serum level of FGF19 was found in lung cancer patients, which could also serve as a novel diagnostic index to screen lung cancer. Overproduction of FGF19 in LSQ cells markedly promoted cell growth, progression and metastasis, while downregulating FGF19 effectively inhibited LSQ progression in vitro and in vivo. Moreover, downregulating the receptor FGFR4 was also effective to suppress the growth and migration of LSQ cells. Since FGF19 could be induced by smoking or endoplasmic reticulum stress, to tackle the more malignant FGF19-overproducing LSQ, we reported for the first time that inhibiting mTOR pathway by using AZD2014 was effective and feasible. These findings have offered a new strategy by using anti-FGF19/FGFR4 therapy or mTOR-based therapy in FGF19-driven LSQ.

## Introduction

Lung cancer is the most common type of cancer and the leading cause of cancer death in China and around the world. The 5-year survival rate of lung cancer is merely 19% since more than 50% of the cases are diagnosed at an advanced stage [1, 2]. With the impact of smoking and air pollution, the burden of lung cancer in China may continue to rise in the coming decades [1]. Lung squamous cell carcinoma (LSQ) is the second most common type of lung cancer after adenocarcinoma among non-small cell lung cancer (NSCLC). It accounts for nearly 30% of NSCLC, in which over 90% are smoking patients [3]. Recently, targeted therapies against specific genes including EGFR, ALK, ROS1, and BRAF have markedly improved the outcome for adenocarcinoma patients [4]. However, only a small proportion of LSQ harbor these driver gene mutations. Therefore, it is urgent to further address new driver genes of LSQ for the development of more effective treatment options.

Fibroblast growth factors (FGF) interact with four receptor tyrosine kinases (FGFR1–4) to induce receptor dimerization and autophosphorylation, ultimately resulting in the activation of various signal transduction cascades [5, 6]. Our group has previously identified FGF19 as a prognostic marker and potential driver gene of LSQ in Chinese smoking patients [7]. FGF19, functioning as an endocrine factor, is important in regulating metabolism under normal conditions, yet it could be critical for the progression of several cancers. In patients with hepatocellular carcinoma, high amplification of FGF19 promotes progression and epithelial-mesenchymal transition (EMT) by modulating the GSK3β/β-catenin or STAT3 pathway [8–11]. FGF19 also protects hepatocellular carcinoma cells against endoplasmic reticulum (ER) stress [12]. Furthermore, FGF19 is critical for the progression of breast cancer [13], prostate cancer [14, 15], head and neck squamous cell carcinoma [16] and thyroid cancer [17]. An anti-FGF19 monoclonal antibody 1A6 acting to block the interaction of FGF19 with its receptor FGFR4 could inhibit the colon tumor xenografts in vivo [18].

FGFR4, the direct receptor of FGF19 and predominately expressed in liver tissues, was reported to be highly expressed in oral and oropharyngeal squamous cell carcinoma with a frequent Gly388Arg SNP [19]. Similar findings were reported in intrahepatic cholangiocarcinoma [20], colorectal cancer [21], and hepatocellular carcinoma [22]. Mutated FGFR4 was found to induce local growth and enhance metastasis in these cancers [23]. In breast cancer, FGFR4 resist cancer cell apoptosis by phosphorylating MST1 [24]. Hence, FGFR4, owing to the oncogenic activity, may serve as a potential predictive biomarker for anti-FGFR4 targeted therapy [25]. Thereby, the FGF19/FGFR4 axis is a potential therapeutic target in cancers. However, the molecular mechanisms of how a surge of FGF19/FGFR4 could regulate the progression of LSQ have not been explored in detail.

The FGF19/FGFR4 axis has also been reported to induce mTORC1 and ERK pathways to converge on S6 in hepatoma cells and in head and neck squamous cell carcinoma (HNSCC) [16, 26]. Several PI3K/AKT/mTOR pathway inhibitors have shown antitumor activity in preclinical models of NSCLC [27]. AZD2014, a small molecular drug currently in phase II clinical trials, could block signals from both mTORC1 and mTORC2 complexes in ER + breast cancer [28]. Furthermore, combined treatment with mTOR inhibitors and FGFR-specific tyrosine kinase inhibitors exhibited clinical efficacy for FGFR1-driven lung cancers and HNSCC [29]. However, the involvement of mTOR in FGF19 signaling in LSQ is not fully explored.

In this study, we have revealed that FGF19 is an oncogenic driver in LSQ development and progression, while mTOR plays an essential role in FGF19-driven LSQ. Inhibitors targeting FGF19/FGFR4 axis or the mTOR kinase are effective therapeutics to suppress LSQ.

## Results

### FGF19 is upregulated in LSQ tissues and is a poor prognostic marker in LSQ

**Fig. 1:**
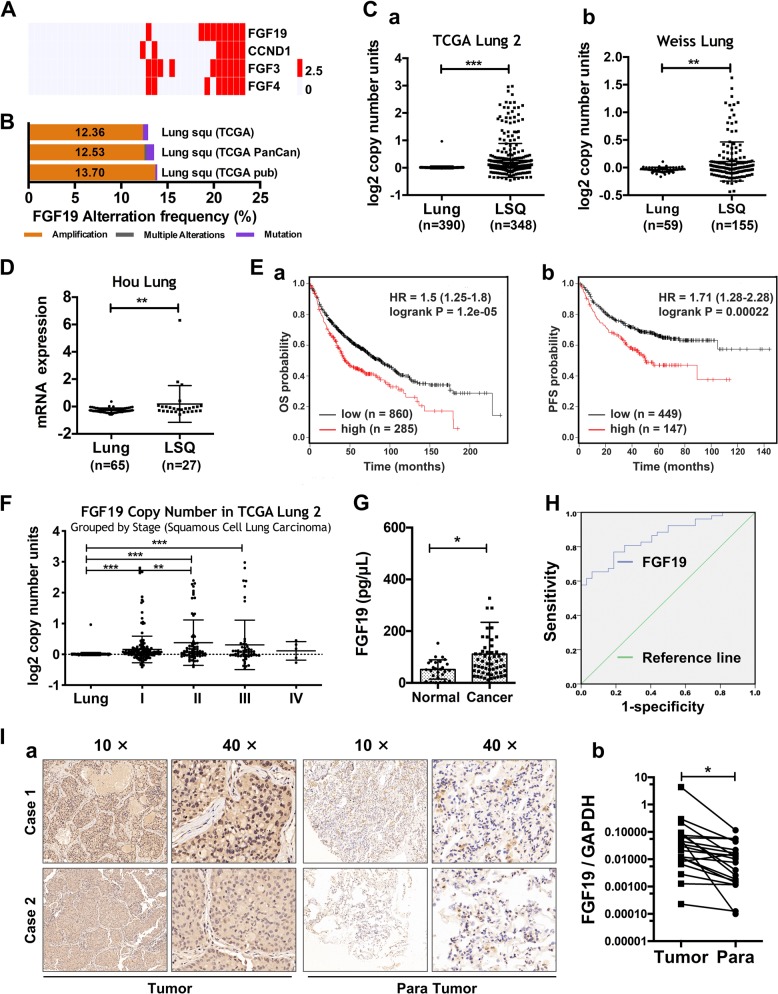
FGF19 is amplified and overexpressed in patients with LSQ. **a** Copy number variation (CNV) distribution of FGF19 in our LSQ cohort (*n* = 37). Mutations including 5 missense mutations (2 duplicate mutation): R151L, P191L, P199A; 1 truncating nonsense mutation: G205* and 1 fusion: ANO1-FGF19. **b** RNAseq expression analysis of FGF19 showing significantly high amplification in TCGA LSQ cases. **c** Copy number analyses of FGF19 in the Oncomine database with provisional LSQ cohorts in TCGA Lung2 datasets (a) and Weiss Lung datasets (b). **d** Expression of FGF19 in the Oncomine database with provisional LSQ cohorts in Hou Lung datasets. **e** Kaplan–Meier survival analysis based on RNAseq expression of FGF19 (higher FGF19 level: top 25%, lower FGF19 level: bottom 25%) on the overall survival (OS; *n* = 1145) (a) and progression-free survival (PFS; *n* = 596) (b) in lung cancer patients (http://www.kmplot.com). **f** Copy number of FGF19 grouped by stage of samples in TCGA Lung2 datasets from the Oncomine database with provisional LSQ cohorts. **g** Serum levels of FGF19 in NSCLC patients (*n* = 57) were significantly higher than in control subjects (*n* = 27). **h** Receiver operating characteristic (ROC) curve analysis of serum FGF19 levels in 57 NSCLC patients vs. 27 controls (*p < 0.05). **i** Representative IHC results for FGF19 expression in LSQ tissue arrays (a) and quantitative data of staining intensity presented as positive area score (b). *p < 0.05; **p < 0.01; ***p < 0.001.

FGF19 is highly amplified in lung squamous cell carcinoma, which ranks the fourth in the frequency in all examined cancer types from the TCGA Pan-Cancer cohort [16]. By quantitative RT-PCR analysis, we observed in our cohort [7] that FGF19 gene copy number increased in 9 out of 37 samples, along with increased copy number of three other genes CCND1, FGF3 and FGF4 (Fig. 1a). This increase is also corresponded with the amplification of FGF19 in mRNA expression (http://www.cbioportal.org) in LSQ (Fig. 1b). Meanwhile, we analyzed copy number and expression features of FGF19 in the LSQ cohort from the Oncomine database. Copy number of FGF19 was increased compared with the normal lung tissues in TCGA Lung2 datasets (Fig. 1Ca) and in Weiss Lung datasets (Fig. 1Cb). Consistently, FGF19 mRNA expression was increased in Hou Lung datasets (Fig. 1d) in LSQ cohorts. We then analyzed the Kaplan–Meier plots and found that higher FGF19 level (top 25%) was associated with shorter overall survival (OS, *n* = 1145) (Fig. 1Ea) and progression-free survival (PFS, *n* = 596) (Fig. 1Eb) vs. lower FGF19 level (bottom 25%) in NSCLC. We also found a high copy number of FGF19 in TCGA Lung2 datasets respectively by stage (Fig. 1f). To verify whether serum levels of FGF19 in lung cancer patients corresponded with the observations of high CNV and mRNA expression from the database, we further analyzed clinical samples. Expectedly, serum levels of FGF19 in NSCLC patients were significantly higher than those in control subjects (Fig. 1g). Moreover, from receiver operating characteristic (ROC) curve analysis, we calculated that when the serum level of FGF19 was higher than 65 ng/mL, the sensitivity was 75% and the specificity was 81.2% (Fig. 1h). IHC analysis showed significantly higher levels of FGF19 in LSQ tissues compared with paired neighboring noncancerous tissues (Fig. 1i). These findings further indicated that FGF19 is upregulated and contributes to cancer development and progression in LSQ.

### CDCA and ER stress induces FGF19 upregulation and elevated FGF19 activates ERK/AKT signaling to promote cell proliferation

**Fig. 2:**
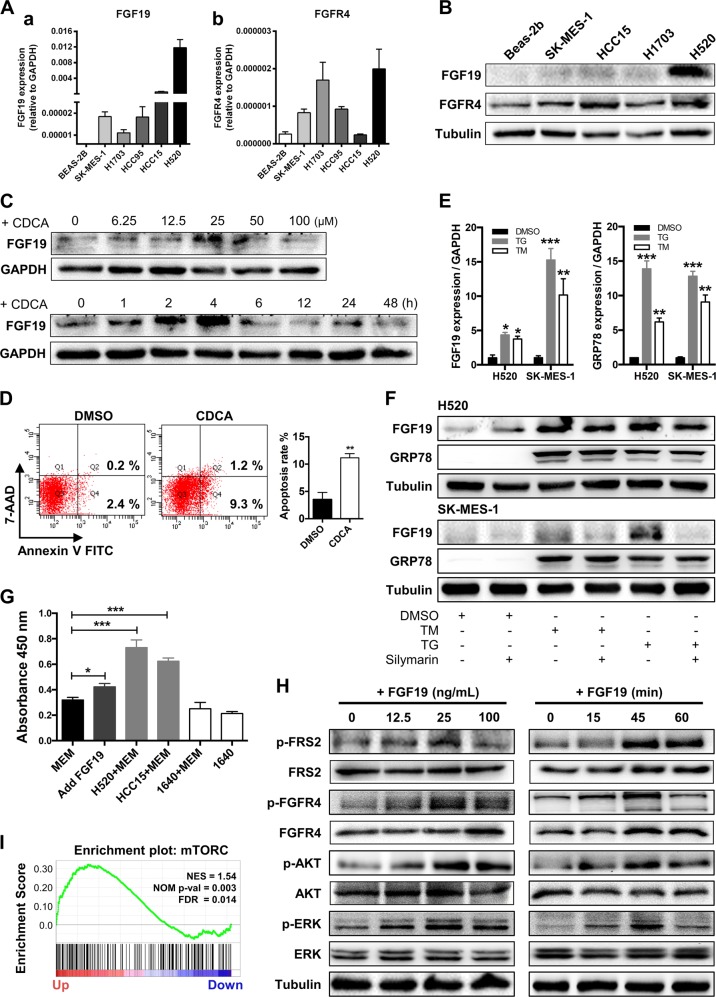
CDCA and ER stress induce FGF19 upregulation and hFGF19 activates ERK/AKT signaling to promote LSQ cell proliferation. **a** FGF19 (a) and FGFR4 (b) mRNA expression levels in LSQ cell lines and Beas-2b. **b** Protein expression levels of FGF19 and FGFR4 in LSQ cell lines and bronchial epithelial cell line Beas-2b. **c** The effects of chenodeoxycholic acid (CDCA) treatment on FGF19 protein levels in Beas-2b cells in a dose-dependent (ranging from 0 to 100 µM, upper panel) and time-dependent (within 48 h, lower panel) manner. **d** The effect of chenodeoxycholic acid treatment (50 µM) on the apoptosis of Beas-2b cells with quantifications of the results on the right panel. **e** mRNA expression of FGF19 (left panel) and GRP78 (right panel) of LSQ cell lines H520 and SK-MES-1 treated with thapsigargin (TG) or tunicamycin (TM) for 24 h. **f** FGF19 protein expression of H520 and SK-MES-1 treated with TG or TM for 24 h, in the presence or absence of ER stress suppressor silymarin. **g** Cell proliferation of SK-MES-1 cells treated with conditioned medium from H520 cells overexpressing FGF19 was determined by CCK-8 assays. **h** The effects of hFGF19 on the activation of ERK/AKT signaling in SK-MES-1 cells in a dose-dependent (left panel) or time-dependent (right panel) manner. **i** Enrichment plots of mTOR expression signatures according to FGF19 expression levels in an LSQ cohort.

We then analyzed the role of FGF19 in LSQ cell lines. The basal expression of FGF19 in these LSQ cell lines and a normal bronchial epithelial cell line Beas-2b were determined. In agreement with our clinical findings, expression of FGF19 was upregulated in these LSQ cell lines, both in mRNA (Fig. 2Aa) and protein (Fig. 2b) levels. Extremely high expression of FGF19 in H520 and HCC15 cells was in accordance with data from the CCLE database among 186 cell lines (Supplementary Fig. 1A). Consistently, FGFR4, as the main receptor of FGF19, was also upregulated in these LSQ cell lines (Fig. 2Ab and Fig. 2b).

Next, we explored which factor induced the elevated expression of FGF19. In Beas-2b cell lines, chenodeoxycholic acid (CDCA) was reported to increase FGF19 expression [30]. Here, again we observed elevated FGF19 protein expression time- and dose-dependently (Fig. 2c) along with elevation in the proportion of apoptotic cells (Fig. 2d). We also found that treatment of ER stress inducers thapsigargin (TG) and tunicamycin (TM) led to a significant increase in FGF19 expression in H520 and SK-MES-1 cell lines, together with an increase of the ER stress marker GRP78 in mRNA levels (Fig. 2e). We also treated H520 and SK-MES-1 cells with silymarin, an ER stress repressor, in the presence of TG or TM. Decreased expression of FGF19 was found in the co-treatment group, as compared with those with TG or TM single drug treatment (Fig. 2f) in H520 and SK-MES-1 cells.

Thereafter we determined the potential role of elevated FGF19 in LSQ cells. In SK-MES-1, H1703 and HCC15 cells, hFGF19 significantly promoted cell proliferation (Supplementary Fig. 1B). Given that FGF19 is a secreted protein, we added conditioned medium of H520 cells containing high levels of FGF19 to SK-MES-1 cells, and observed an increased proliferation (Fig. 2g). Moreover, hFGF19 activated both ERK and AKT signaling in SK-MES-1 cells time- and dose-dependently (Fig. 2h). We also detected this activation in H520 and HCC15 cells (Supplementary Fig. 1C). Furthermore, GSEA plots for FGF19 in LSQ cohorts revealed that mTOR signaling was enriched in FGF19 up genes (Fig. 2i), accompanied by cell division, DNA replication, WNT signaling and other cancer-related pathways such as VEGF-A, MYC, SHH signaling and ATF4, which is a critical ER stress-inducible transcription factor, while TGF-beta and P53 were enriched in FGF19 down genes (Supplementary Fig. 2 and Supplementary Table 1). Together these results indicated that FGF19 was upregulated after CDCA stimulation or ER stress, and elevated FGF19 activated ERK/AKT signaling to promote LSQ cell proliferation.

### Overexpression of FGF19 promotes the proliferation of LSQ cells in vitro and in vivo

Since there was a correlation between the expression of FGF19 and LSQ progression and elevated FGF19 promoted cell proliferation, we further explored the functional role of FGF19 in LSQ. We first constructed the FGF19 overexpressing vector (FGF19-OE) which was also packaged into a lentiviral system. After stable infection of FGF19-OE lentivirus into SK-MES-1 and HCC95 cells, which are two relatively not high FGF19-amplified LSQ cell lines, the effects of FGF19 activity on proliferation were measured.

**Fig. 3:**
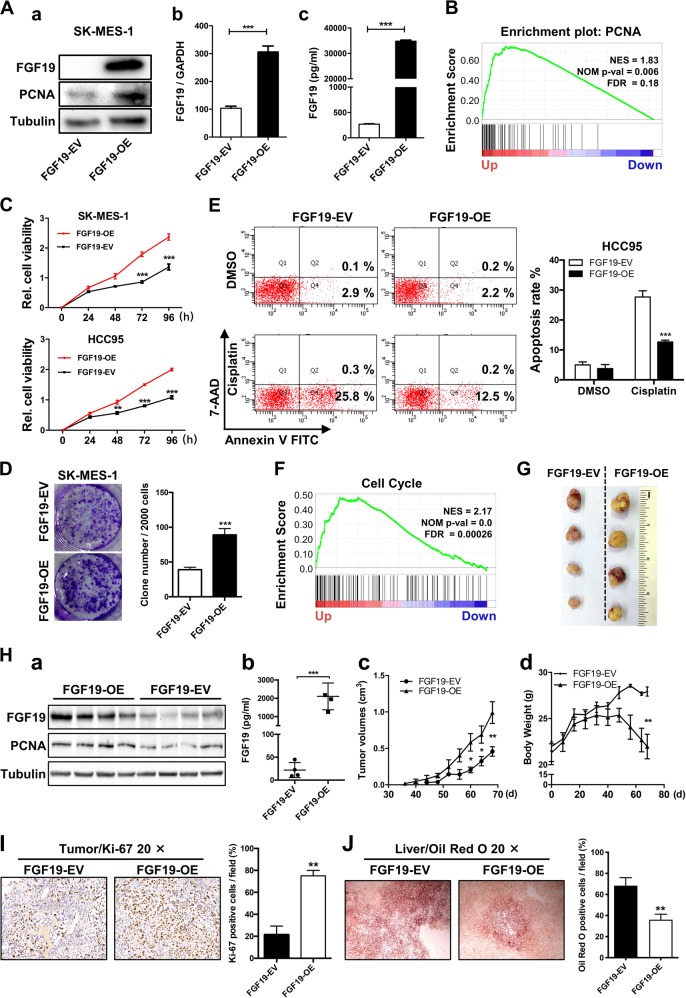
Overexpression of FGF19 promotes the proliferation of LSQ cells in vitro and in vivo. SK-MES-1 and HCC95 cells were transduced with FGF19 overexpression lentivirus (FGF19-OE), or control lentivirus (FGF19-EV) to construct stable cell lines. **a** Quantification of the overexpression of FGF19 in forms as cellular protein (a), mRNA (b) or secreted protein in conditioned medium (c) in SK-MES-1 cells. **b** Enrichment plots of PCNA expression signatures according to FGF19 expression levels in an LSQ cohort. **c** Cell proliferation assays of the SK-MES-1 and HCC95 stable cells with FGF19 overexpression. **d** Colony formation in SK-MES-1 cells with or without FGF19 overexpression. **e** The effect of overexpression of FGF19 on the apoptosis induced by cisplatin in HCC95 cell lines. **f** Enrichment plots of cell cycle signatures according to FGF19 expression levels in an LSQ cohort. **g** Images of tumor nodules from subcutaneous mouse xenograft model with or without FGF19 overexpression. **h** (a) Western blot analysis of FGF19 and PCNA expression in tumors; (b) serum samples of FGF19 expression; (c) volume of tumors; and (d) body weight of mice from the two groups. **i** IHC analysis of Ki-67 expression in tumors. **j** Oil red O analysis of tumors from the two groups. Data are represented as mean and SEM from three independent experiments. *p < 0.05; **p < 0.01; ***p < 0.001.

In the stably transfected SK-MES-1 cells, FGF19 was highly expressed at both protein and mRNA levels, along with elevation in both PCNA expression and secreted FGF19 protein (Fig. 3a). This was in line with the results from GSEA plots for FGF19 in LSQ cohorts that PCNA was enriched in FGF19 up genes (Fig. 3b) and a significant increase in cell growth was found in SK-MES-1 and HCC95 cell lines (Fig. 3c). Moreover, FGF19 overexpression led to a significant increase in colony formation by about 50% (Fig. 3d). Meanwhile, overexpression of FGF19 resisted the apoptosis induced by cisplatin in the HCC95 and SK-MES-1 cell line (Fig. 3e and Supplementary Fig. 3) and cell cycle-related genes were found in FGF19 up genes from GSEA plots for FGF19 in LSQ cohorts (Fig. 3f).

The in vitro data prompted us to investigate the role of FGF19 in LSQ in vivo. Using a subcutaneous model, a marked increase in tumor size was observed in mice receiving FGF19-OE lentivirus compared with those receiving the control lentivirus in SK-MES-1 cells (Fig. 3g). This effect was also accompanied by higher PCNA expression, higher serum FGF19 levels, increased tumor volume and decreased body weight in the FGF19-OE group vs. control group (Fig. 3h). IHC results revealed that the levels of Ki-67 were significantly increased in the mice injected with FGF19-OE cells compared with the mice receiving the vehicle control cells (FGF19-EV) (Fig. 3i). We further performed Oil red O staining of tumors from these two groups and observed that intracellular lipid content in FGF19-OE groups was reduced (Fig. 3j). To summarize, these observations demonstrated that FGF19 could promote the proliferation of LSQ cells in vitro and in vivo.

### Overexpression of FGF19 promotes metastasis of LSQ cells in vitro and in vivo

**Fig. 4:**
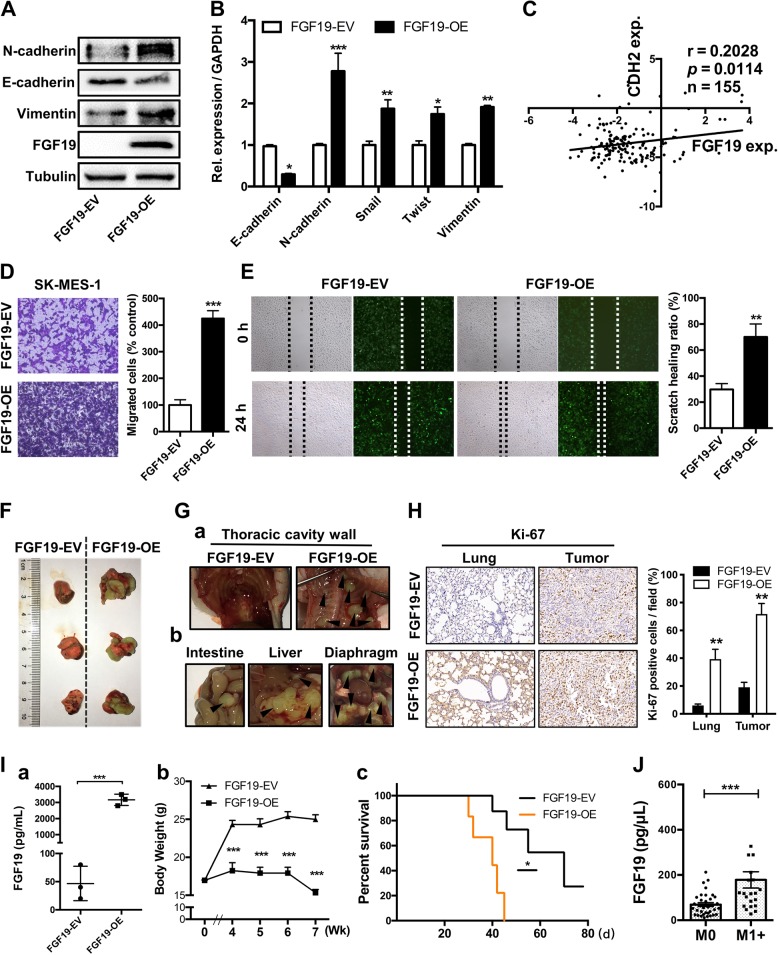
Overexpression of FGF19 promotes metastasis of LSQ cells in vitro and in vivo. **a** Protein and **b** mRNA expression of EMT markers in the cells treated with FGF19-EV or FGF19-OE for 48 h in SK-MES-1 cells. **c** Analysis of correlation between CDH2 and FGF19 from Oncomine TCGA datasets. The images of migration abilities in FGF19-OE transfected cells in trans-well (**d**) and wound healing (**e**) assays, respectively. **f** Overexpression of FGF19 increased tumor growth in vivo in orthotopic lung cancer models. Representative image of primary tumors in the left lungs of orthotropic models on day 21 from each group after implantation of SK-MES-1 cells transfected with FGF19-OE and FGF19-EV. **g** Metastatic tumors in other organs. **h** Representative images of IHC analysis of Ki-67 expression in lung and tumors. Right panel: Quantification of Ki-67 expression. **i** (a) Evaluation of FGF19 expression in serum samples from two groups. (b) Mean primary tumor body weight and (c) survival curve for the mice in each treatment group evaluated. **j** Within the 57 NSCLC patients’ group, the serum levels of FGF19 in patients with metastasis (*n* = 34) were shown side by side with those of patients without metastasis (*n* = 23). Data are represented as mean and SEM from three independent experiments. *p < 0.05; **p < 0.01; ***p < 0.001.

We next examined whether FGF19 could play a role in tumor metastasis. We observed that upregulation of FGF19 increased mesenchymal markers N-cadherin and Vimentin, and reduced epithelial marker E-cadherin, in both protein levels (Fig. 4a) and mRNA expression (Fig. 4b). Meanwhile, from the Oncomine TCGA database, we found a positive relationship between N-cadherin (CDH2) and FGF19 (Fig. 4c). In SK-MES-1 cells, the migratory ability was significantly increased in FGF19-OE transfected cells as revealed in trans-well (Fig. 4d) and wound healing (Fig. 4e) assays.

To verify the role of FGF19 in a physiologically more relevant tumor background, we developed an orthotopic mouse model to closely recapitulate the clinical features of human lung cancer by using SK-MES-1 cells that were stably infected with FGF19-OE lentivirus or control lentivirus FGF19-EV (*n* = 10 per group). Compared with the FGF19-EV group, overexpression of FGF19 significantly increased the progression of tumors (Fig. 4f) and more metastatic nodules were detected in the FGF19-OE group (Fig. 4g). IHC analysis of Ki-67 expression revealed that a remarkable increase of proliferation in tumor and lung of the FGF19-OE groups (Fig. 4h), along with high serum levels of FGF19 (Fig. 4Ia). This effect was also accompanied by decreased mice body weight (Fig. 4Ib) and shorter overall survival (Fig. 4Ic) in the FGF19-OE group vs. control group. Moreover, serum levels of FGF19 in patients with metastasis (*n* = 34) were higher than patients without metastasis (*n* = 23) (Fig. 4j). Altogether, these results indicated that the upregulation of FGF19 could promote the migration, invasion, and EMT of LSQ cells in vitro and in vivo.

### Downregulation of either FGF19 or FGFR4 impairs the malignant phenotypes of LSQ cells

**Fig. 5:**
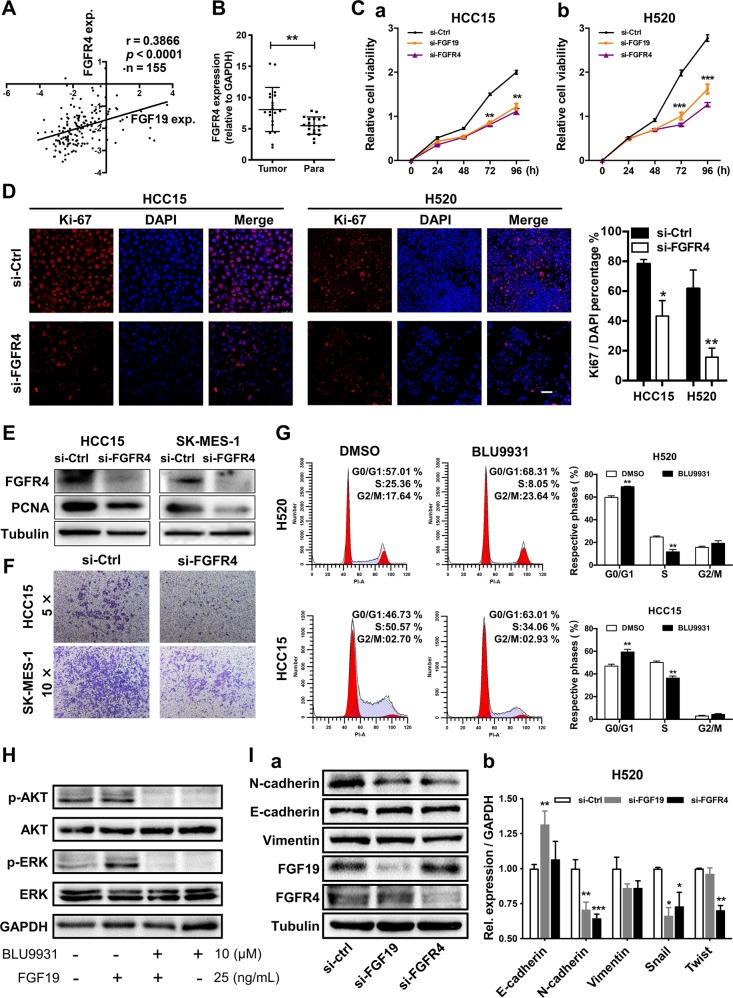
Downregulation of either FGF19 or FGFR4 suppressed the malignant phenotypes of LSQ cells. **a** Analysis of correlation between FGFR4 and FGF19 from Oncomine TCGA datasets. **b** FGFR4 mRNA levels were determined by q-RT-PCR in 20 LSQ samples relative to the matched non-tumor tissues (normalized to GPADH). **c** HCC15 (a) and H520 (b) cells were transfected with either control siRNA, FGF19 siRNA or FGFR4 siRNA. CCK-8 assays were conducted to access cell proliferation. **d** Immunofluorescence staining of Ki-67 (red) and DAPI (for nucleus, blue) in HCC15 and H520 cells after siRNA transfection. Scale bar = 50 µm. The right panel was the percentage of Ki-67 positive cells. **e** Protein levels of FGFR4 and proliferation marker PCNA were determined by western blot. **f** Downregulation of FGFR4 suppressed cell migration via trans-well assays in HCC15 and SK-MES-1 cells; **g** Representative histograms depicting cell cycle profiles of H520 and HCC15 cells with or without FGFR4 inhibitor BLU9931. Right panel: Quantifications of the histograms. **h** Inhibition of FGFR4 attenuates FGF19- mediated ERK/AKT signaling in H520 cells. **i** (a) Protein and (b) mRNA expression of EMT markers in the cells transfected with either control siRNA, FGF19 siRNA or FGFR4 siRNA for 48 h in H520 cells. Data were collected from three independent experiments. *p < 0.05; **p < 0.01; ***p < 0.001.

To confirm whether FGF19 signals through its receptor FGFR4 to exert cellular functions [31], we first searched in the Oncomine database and found that FGFR4 and FGF19 showed a positive relationship in TCGA datasets (Fig. 5a). To explore the relationship between FGFR4 expression levels and LSQ progression, we examined the FGFR4 expression in 20 pairs of LSQ samples. FGFR4 mRNA levels were higher when compared with the matched non-tumor tissues (Fig. 5b) and western blot analysis showed increased FGFR4 expression in LSQ samples (Supplementary Fig. 4A). We then transfected cells with either control siRNA, FGF19 siRNA or FGFR4 siRNA, using two relatively high FGF19-amplified LSQ cell lines, H520 and HCC15. In these two cell lines, cell growth was significantly inhibited (Fig. 5c), and immunofluorescence Ki-67 signals were significantly reduced by either FGF19 or FGFR4 knockdown (Fig. 5d). Direct FGFR4 siRNA transfection effectively decreased FGFR4 protein levels, accompanied by a significant reduction of PCNA level (Fig. 5e) and cell migration (Fig. 5f) in HCC15 and SK-MES-1 cells. Accordingly, by FGF19 siRNA transfection, H520 cells showed a significant reduction of PCNA level (Supplementary Fig. 4B). To further evaluate the function of FGFR4 in FGF19 signaling, we treated H520 and HCC15 cells with the FGFR4-specific inhibitor BLU9931 [32] and observed that BLU9931 induced G1 to S phase arrest (Fig. 5g) and significantly abolished FGF19-mediated induction of ERK and AKT signaling (Fig. 5h and Supplementary Fig. 4C). Similar results were obtained by using another pan FGFR inhibitor BGJ398 (Supplementary Fig. 4D). Moreover, either FGF19 siRNA or FGFR4 siRNA transfection in H520 cells enhanced the sensitivity to Taxol or cisplatin (Supplementary Fig. 4E). Loss of FGF19 or FGFR4 more or less reversed the expression pattern of EMT markers (Fig. 5i and Supplementary Fig. 4F) as observed in the FGF19 overexpression experiment (Fig. 4a), and decreased expression of stemness-related genes (Supplementary Fig. 4G) in H520 and HCC15 cells. Collectively, these findings demonstrated that loss of either FGF19 or FGFR4 could impair the malignant phenotypes of LSQ cells.

### mTOR inhibitor AZD2014 suppresses growth of FGF19-overproducing LSQ cells in vivo

**Fig. 6:**
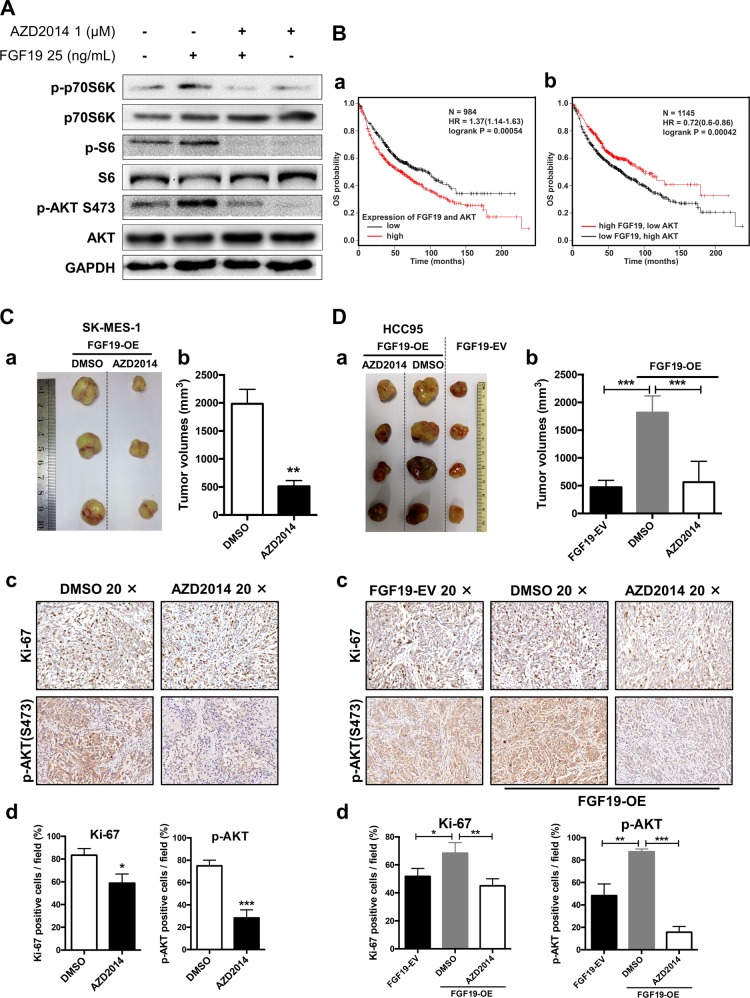
mTOR inhibitor AZD2014 suppresses tumor growth of LSQ cells with FGF19 overexpression in vivo. **a** Western blot analysis shows mTOR pathways inhibited by AZD2014. SK-MES-1 cells were stimulated with FGF19 (25 ng/mL) for 20 min with or without pretreatment of AZD2014 (1 μM) for 40 min. **b** (a) Higher FGF19 and AKT mRNA levels (FGF19: AKT = 1:1) are associated with shorter overall survival, evaluated by Kaplan–Meier Plotter (http://www.kmplot.com). (b) Higher FGF19 and lower AKT mRNA levels are associated with longer overall survival. **c** Representative images (a) and quantitative data (b) of in vivo subcutaneous lung cancer models. FGF19-OE tumors of SK-MES-1cells were re-transplanted subcutaneously, treated with 15 mg/kg (2 days on/5 days off) of AZD2014 or equivalent amount of DMSO vehicle by intragastric administration for 3 weeks. IHC analysis of Ki-67 and p-AKT(S473) expression in tumors (c) with quantitative data (d). **d** Images of tumor nodules from subcutaneous mouse xenograft model with or without FGF19 overexpression in HCC95 cells treated with AZD2014 or DMSO for 3 weeks in FGF19-OE group from the first week (a) with quantitative data (b). IHC analysis of Ki-67 and p-AKT(S473) expression in tumors (c) with quantitative data (d).

In hepatocellular carcinoma cells, the FGF19/FGFR4 axis has been shown to upregulate ERK/AKT–p70S6K–S6 pathway [26]. We have also examined that ERK/AKT pathway was upregulated when FGF19 was overexpressed in this study. Thus, it is important to determine the function of these signaling events in vivo. As shown in Fig. 2h, mTOR signaling was enriched in FGF19 up genes, thus we selected AZD2014, an ATP-competitive inhibitor of mTOR, to perform in vivo experiments. In vitro, AZD2014 significantly inhibited AKT–p70S6K–S6 pathway in SK-MES-1 cells (Fig. 6a and Supplementary Fig. 5A). Higher FGF19 and AKT mRNA levels (top 25%) were associated with shorter overall survival (Fig. 6Ba), and such association was also observed in groups of higher FGF19 combined with higher FGFR4, MYC and GLI1 expression (Supplementary Fig. 5B). However, higher FGF19 and lower AKT mRNA levels were associated with longer overall survival (Fig. 6Bb). These results prompted us to investigate whether inhibition of AKT in FGF19-overexpressed cells have any effect in inhibiting cancer cell growth.

We first generated subcutaneous lung cancer models with tumors from experiments shown in Fig. 3g (FGF19-OE), and treated mice with AZD2014 or an equal volume of vehicle by intragastric administration for 3 weeks [28]. In this model, a marked reduction in tumor growth was observed in mice treated with AZD2014 (Fig. 6Ca, b), along with a decreased expression of Ki-67 and p-AKT (Fig. 6Cc, d). We also detected the same results in another mouse model using HCC95 cells (Fig. 6d).

To determine whether downregulating FGF19 combined with AZD2014 has a synergistic effect, we tested in subcutaneous mouse models using HCC15 or H520 cells that were stably transfected with sh-CTRL or sh-FGF19 lentivirus. We found that downregulation of FGF19 inhibited the progression of tumor cells, whereas administration of AZD2014 did not further increase this inhibitory effect, both in tumor volume and Ki-67, p-AKT expression levels (Supplementary Fig. 6).

In conclusion, AZD2014 could effectively suppress the growth of FGF19- overexpressing LSQ cells in vivo.

## Discussion

As a follow-up to our previous report that revealed an amplified FGF19 in smoking LSQ patients, in this study, we did further research on the critical role of the oncogenic driver FGF19 in LSQ and explored the potential therapeutic target such as mTOR in FGF19-overproducing LSQ.

The present study provides evidence that FGF19, being highly amplified and expressed, plays an essential role in tumorigenesis in LSQ. Moreover, CDCA or ER stress-induced FGF19 upregulation in Beas-2b cells and LSQ cells. Patients with esophageal reflux may be more likely to suffer an increase in FGF19, and all the factors that induced ER stress may facilitate the upregulation of FGF19 and eventually leads to a vicious circle, since increased FGF19, in turn, will promote tumor progression. This is in accordance with results reported that ER acts as a moving organelle with many important cellular functions [33] and FGF19 was reported to protect hepatocellular carcinoma cells against ER stress [12]. Our study established an FGF19-overproducing model to mimic the more malignant phenotype of FGF19-overexpressed LSQ. In addition to the downregulation of FGF19 to demonstrate the anti-cancer effect that was also shown in other cancer models, herein we demonstrated for the first time that overexpressing FGF19 promoted tumor cell proliferation and metastasis in vivo in LSQ. Moreover, knockdown of FGFR4 was also effective to suppress the growth and migration of LSQ cells. Thus, inhibitors specific to the FGF19/FGFR4 axis or antibodies for FGF19 or FGFR4 may exert an antitumor effect in FGF19/FGFR4 driven LSQ patients. On the other hand, directly targeting FGF19 in LSQ might not be very effective since FGF19 could be highly expressed during cancer progression. To resolve this issue, we analyzed the datasets and association of key genes, and identified mTOR as a good candidate target. Indeed, we discovered that inhibiting mTOR in FGF19-overproducing LSQ resulted in significant tumor inhibition. This finding has clinical implications and should be followed in future studies.

For LSQ cells with basal FGF19 levels, downregulating FGF19 combined with mTOR inhibitor AZD2014 showed no significant difference from downregulating FGF19 alone groups in the effective tumor inhibition in vivo. In this scenario, knockdown FGF19 is potent enough to suppress the growth of LSQ cells since mTOR is a direct downstream effector of the FGF19 signal. However, this result is slightly different from an earlier report in which downregulating FGF19 alone could not suppress tumor formation in LSQ [34]. This could be explained by the different basal levels of FGF19 in the different cell lines. As we have shown in Supplementary Fig. 1A, the basal expressions of FGF19 in H520 and HCC15 cell lines are both relatively high, while in the reported article, EPLC272H was used as a high-expressed cell line but its FGF19 expression was lower than H520 and HCC15.

Beta-klotho, reported as a co-receptor of FGFR4, is required for high-affinity binding and activity of FGF19 in specific liver functions [35]. It was reported that beta-klotho, in partnership with FGFR4, induced cell apoptosis and inhibited cell proliferation in liver cancer [36]. The mechanism of beta-klotho in NSCLC seems to be complicated and we have reported our findings in a separate study, in which we found that beta-klotho inhibited NSCLC progression [37]. Compared with non-tumor tissues, there was a downregulation of beta-klotho in NSCLC, and such a lowered beta-klotho level could increase cell proliferation and metastasis. Furthermore, beta-klotho overexpression inhibited NSCLC tumor growth in vitro and in vivo. Nonetheless, future studies are needed to fully examine the role of beta-klotho in FGF19 induced cell proliferation in LSQ.

Our findings have several notable implications. First, serum levels of FGF19 were significantly upregulated in NSCLC and especially upregulated in patients with metastasis vs. patients without metastasis. Thus, high expression of FGF19 may serve as a novel diagnostic index for screening LSQ, although a larger sample size is needed in future studies. Second, higher expression of FGF19 in NSCLC exhibited shorter OS and PFS in patients with NSCLC. Third, downregulation of FGF19 inhibited LSQ progression and it may achieve therapeutic potential. Further, inhibition of the mTOR pathway performed a good outcome in FGF19-driven LSQ. Hence, FGF19 is identified as a driver gene in LSQ. Collectively, the current study has proposed this new role of FGF19 as a target for LSQ, and in FGF19-overproducing LSQ, mTOR is a promising therapeutic target.

In conclusion, we have demonstrated the oncogenic activity of FGF19 in LSQ. Our findings have also implicated the values of using anti-FGF19/FGFR4 therapy or mTOR-based therapies in the treatment of FGF19-driven LSQ.

## Material and methods

### Clinical tissue specimens and serum samples

NSCLC serum samples including 57 from patients and 27 from normal subjects and human LSQ and their matched non-tumor tissue samples (20 pairs) were obtained from Shanghai Chest Hospital from January to November 2017. All the clinic pathological characteristics of samples were summarized in our previous work [37] following protocols approved by the institute research ethics committee of School of Biomedical Engineering, Shanghai Jiao Tong University.

### Cell lines, reagents, and antibodies

Beas-2b, SK-MES-1, HCC15, H1703, H520 and HCC95 cell lines were obtained from ATCC. Exogenous FGF19 was purchased from R&D (#969-FG-025). Chenodeoxycholicacid, thapsigargin, tunicamycin, silymarin, DMSO, DAPI, and PEG were purchased from Sigma-Aldrich. FGFR4 inhibitor BLU9931 and FGFR pan inhibitor BGJ398 were purchased from Selleck. mTOR inhibitor AZD2014 was kindly offered by AstraZeneca Pharmaceutical Company. FGF19(#AF969) and FGFR4(#MAB6852) antibodies were purchased from R&D. Tubulin (#ab179513), Ki-67 (#ab92742) and FRS2 (#ab10425) antibodies were purchased from Abcam and other antibodies were obtained from Cell Signaling Technology.

### siRNA and plasmids transfection

FGF19 siRNA were purchased from Dharmacon (#L-013670) and FGFR4 siRNA were purchased from Life technologies. FGF19 RNAi plasmids were constructed by GENECHEM. The full-length FGF19 gene was amplified from ORF plasmids (OriGene: #RC203750) and cloned into pCDH expression vector (Addgene plasmid: #72265) to constructed plasmid for lentivirus production. Plasmids or siRNAs were transfected by Lipofectamine 3000 (Invitrogen) following the manufacturers’ instructions. Sequences are as follows: siFGFR4, sense 5′-GGAUGGACAGGCCUUUCAUtt-3′, antisense 5′- AUGAAAGGCCUGUCCAUCCtt -3′; target of shFGF19-1: 5′- TTTCTTCCACTCTCTCATT -3′, target of shFGF19-2: 5′- GTACAAGAACAGAGGCTTT -3′. For in vitro experiments, at least three independent experiments were conducted.

### Lentivirus production, infection, and generation of stable cell lines

FGF19 overexpression lentiviruses were produced by HEK293T cells after transfection with FGF19 overexpression plasmids, accompanied by pCMV-dR8.2/pCMV-VSV-G plasmids. Mature lentiviruses were obtained by ultracentrifugation. FGF19 RNAi lentiviruses were constructed by GENECHEM. Lentivirus transductions and stable cell line generation were performed as previously described [38].

### Western blot and quantitative real-time PCR (qRT-PCR) analysis

Cells were lysed for protein or RNA extraction and then subjected to western blot or qRT-PCR as previously described [38]. The detailed sequence of primers was summarized in Supplementary Table 2.

### Immunohistochemistry (IHC) and Immunofluorescence microscopy (IF)

IHC experiment was conducted as previously described [38]. The stained cells were photographed and five microscopic fields were counted. Briefly, by image analysis software Image J, we analyzed total number of cells and the number of positively stained cells (in the nuclear) in each photo and calculated the percent of positive cells. For IF, cells were seeded on coverslips and immunofluorescence staining was conducted as previously described [38]. Stained cells were photographed under an immunofluorescence microscope (Leica DFC420C).

### Enzyme-linked immunol sorbent assay (ELISA)

Blood samples were processed within 1 h after collection and stored at −80 °C until analysis. Serum concentrations of FGF19 were evaluated using ELISA kits (R&D #DF1900) following the manufacturer’s instructions.

### Flow cytometry-based Annexin V/7-AAD assay

The flow cytometry assay was performed to measure the levels of apoptosis by using ApoScreen Annexin V kit (Southern Biotech, Birmingham, AL, USA) according to the manufacturer’s protocol. The number of stained cells was assessed by a flow cytometer (FACS Aria II, BD Biosciences).

### Cell cycle analysis

Cells were grown to a density of 90% and were fixed with 70% cold ethanol at 4 °C overnight, followed with 100 μg/mL RNaseA for 30 min at 37 °C and 50 μg/mL propidium iodide in the dark. Subsequently, the cells were analyzed by flow cytometry (BD FACS Calibur).

### Colony formation

Cells were infected with either FGF19-OE or FGF19-EV lentiviruses, cultured in media containing G418 for two weeks and stained to determine the number of colonies.

### Cell proliferation assay

Cell Titer 96® Aqueous One Solution Cell Proliferation Assay kit (Promega, USA) was assessed to determine the proliferation rate. Briefly, cells were seeded in a 96-well plate after transfection or in the presence or absence of FGF19 with different concentrations. After 72 h, 10 μL agent a were added and the optical density was measured at 490 nm with a microplate reader (BioTek) after 1–4 h incubation.

### Trans-well migration assay

Cells were serum starved for 24 h after transfection and harvested and re-suspended in serum-free medium in a Trans-well chamber (CORNING, 3422). After incubation, cells were fixed and then stained with 0.1% crystal violet dye. The stained cells were counted at three randomly selected views for subsequent calculations.

### Scratch assay

SK-MES-1 cells were grown to a density of 90% after transfection and were scratched and cultured with fresh medium containing 0.5% serum. Photomicrographs were taken after scratching.

### Oil red O staining

Fresh liver tissues were rapidly frozen and embedded. After fixation in 4% formaldehyde, sections were stained with Oil Red O (Sigma-Aldrich) and counterstained with hematoxylin (Sangon, China). Images were captured using a Leica microscope.

### In vivo subcutaneous lung cancer model

Cell suspension of SK-MES-1, HCC95, HCC15 or H520 (1 × 10^6^ cells) in a volume of 50 μL were injected subcutaneously into 4-week-old male BALB/C nude mice. Tumor volumes were measured every 3 days and calculated as 0.5 × length × width^2^. For the secondary tumor formation experiment, tumors were harvested, cut into 3 mm pieces and implanted into mice subcutaneously (*n* = 5 per group). The mice were randomly grouped and treat with AZD2014, which was diluted in 5 mg/mL with 5% DMSO and 30% PEG 300 in deionized water. After the tumor reached 0.2 cm^3^, the mice were treated with AZD2014 (15 mg/kg, 2 days on/5 days off) or an equal volume of a vehicle for 3 weeks. All procedures were performed following the regulations and internal biosafety and bioethics guidelines of Med-X Research Institute, Shanghai Jiao Tong University.

### In vivo orthotopic lung cancer model

A 5 mm incision was sheared on the dorsal side over the left lung, 0.5 cm below the scapula on the 4-week-old male BALB/C nude mice (*n* = 10 per group). The mice were randomly grouped. Cell suspension of SK-MES-1 (1 × 10^6^ cells) in a total volume of 50 μL (PBS: Matrigel = 4:1) was injected directly into the left lateral lung.

### Analysis of public datasets from oncomine and the use of Kaplan–Meier plotter

Relative mRNA and copy number levels of FGF19, FGFR4, CDH2 of TCGA provisional LSQ cohorts and other LSQ datasets were downloaded from the Oncomine database (https://www.oncomine.org). Prognostic values of FGF19, AKT, FGFR4, MYC, GLI1 mRNA levels were analyzed by Kaplan–Meier survival curves of NSCLC patients, using Kaplan–Meier Plotter (www.kmplot.com) [39].

### Gene set enrichment analysis (GSEA)

GSEA was performed using the Cbioportal database (http://www.cbioportal.org/datasets), in which LSQ datasets (TCGA, PanCancer Atlas) were used. Genes were ranked by the expression of FGF19, then the top and bottom 25% of the genes were chosen to apply analysis in GSEA using KEGG and GO databases following the official user guide of GSEA [40].

### Statistical analysis

All statistical analyses were performed using the GraphPad Prism 5 or SPSS software. Data were expressed as mean ± SD. The paired or unpaired Student’s *t* test or ANOVA were chosen to analyze the statistical significance between two groups and the significance level was set at *p* < 0.05.

## Supplementary information

Supplementary Tables

Supplementary Figure 1

Supplementary Figure 2

Supplementary Figure 3

Supplementary Figure 4

Supplementary Figure 5

Supplementary Figure 6

Supplementary Figure Legends
