# Supplementary material for: Enhanced autocrine FGF19/FGFR4 signaling drives the progression of lung squamous cell carcinoma, which responds to mTOR inhibitor AZD2104
Source: Oncogene. 2020 Feb 28;39(17):3507–21. doi: 10.1038/s41388-020-1227-2 (PMC7176586; doi:10.1038/s41388-020-1227-2)
Supplement: Supplementary file 1 — Supplementary Tables [file 41388_2020_1227_MOESM1_ESM.docx]

**Supplementary Table 1.** **﻿Canonical pathways that were significantly up or down-regulated in high-FGF19-expression LSQ compared with low-expression LSQ**

| GO (UP) | NES | NOM p-val | FDR q-val | GO (DOWN) | NES | NOM p-val | FDR q-val |
| --- | --- | --- | --- | --- | --- | --- | --- |
| Cell division | 2.48 | 0 | 0 | Fatty acid β oxidation | -2.05 | 0 | 0.017 |
| DNA replication | 2.46 | 0 | 0 | Chemokine mediated signaling pathway | -1.92 | 0 | 0.038 |
| DNA recombination | 2.32 | 0 | 0 | Lipid kinase activity | -1.92 | 0 | 0.039 |
| Telomere maintenance | 2.13 | 0 | 0.001 | Bile acid biosynthetic process | -1.85 | 0 | 0.053 |
| Stem cell division | 2.05 | 0 | 0.002 | Regulation of inflammatory response | -1.85 | 0 | 0.052 |
| Stem cell proliferation | 1.99 | 0 | 0.004 | Endoplasmic reticulum | -1.71 | 0.020 | 0.092 |
| Multi organism metabolic process | 1.99 | 0 | 0.004 | Epithelial cell apoptotic process | -1.70 | 0.004 | 0.093 |
| Cell cycle G1 S phase transition | 1.98 | 0 | 0.004 | Regulation of protein secretion | -1.67 | 0 | 0.104 |
| Epithelial to mesenchymal transition | 1.83 | 0 | 0.018 | Lung epithelium development | -1.65 | 0.004 | 0.110 |

**Supplementary Table 2. Real-time PCR primers**

| Gene | Forward primers  （5'～3'） | Reverse primers  （5'～3'） |
| --- | --- | --- |
| FGF19 | CGGAGGAAGACTGTGCTTTCG | CTCGGATCGGTACACATTGTAG |
| FGFR4 | CCATAGGGACCCCTCGAATAG | CAGCGGAACTTGACGGTGT |
| GRP78 | CATCACGCCGTCCTATGTCG | CGTCAAAGACCGTGTTCTCG |
| CyclinD1 | GCTGCGAAGTGGAAACCATC | CCTCCTTCTGCACACATTTGAA |
| SOX2 | GTATCAGGAGTTGTCAAGGC | AGTCCTAGTCTTAAAGAGG |
| CD133 | TCCACAGAAATTTACCTACATTGG | CAGCAGAGAGCAGATGACCA |
| OCT4 | GCAATTTGCCAAGCTCCTGAA | GCAGATGGTCGTTTGGCTGA |
| MYC | CAGCTGCTTAGACGCTGGATTT | ACCGAGTCGTAGTCGAGGTCAT |
| TWIST | GTCCGCAGTCTTACGAGGAG | GCTTGAGGGTCTGAATCTTGCT |
| NANOG | CCTGTGATTTGTGGGCCTG | GACAGTCTCCGTGTGAGGCAT |
| E-cadherin | CGAGAGCTACACGTTCACGG | GGGTGTCGAGGGAAAAATAGG |
| N-cadherin | TTTGATGGAGGTCTCCTAACACC | ACGTTTAACACGTTGGAAATGTG |
| VIMENTIN | GACGCCATCAACACCGAGTT | GACGCCATCAACACCGAGTT |
| SNAIL | TCGGAAGCCTAACTACAGCGA | AGATGAGCATTGGCAGCGAG |
| GAPDH | GGAGCGAGATCCCTCCAAAAT | GGCTGTTGTCATACTTCTCATGG |
