# Supplementary material for: Enhanced autocrine FGF19/FGFR4 signaling drives the progression of lung squamous cell carcinoma, which responds to mTOR inhibitor AZD2104
Source: Oncogene. 2020 Feb 28;39(17):3507–21. doi: 10.1038/s41388-020-1227-2 (PMC7176586; doi:10.1038/s41388-020-1227-2)
Supplement: Supplementary file 8 — Supplementary Figure Legends [file 41388_2020_1227_MOESM8_ESM.docx]

**Supplementary Figure 1. Endogenous FGF19 activates ERK/AKT signaling in cell lines with high FGF19 expression.**

**A.** FGF19 mRNA expression levels in 186 different lung cancer cell lines (data from CCLE database: https://portals.broadinstitute.org/ccle). **B.** The dose-dependent effect of hFGF19 on cell proliferation as determined by CCK-8 assays in LSQ cells. **C.** The effects of hFGF19 on the activation of ERK/AKT signaling in H520, HCC15 cells were shown as a dose-dependent and time-dependent manner.

**Supplementary Figure 2. Gene set enrichment analysis plots for FGF19 in squamous cell lung cancer.**

**A.** FGF19 was among genes activated by ATF4. **B.** FGF19 up-regulated genes were involved in VEGF_A **(a)** and MYC **(b)**. FGF19 down-regulated genes were involved in TGF-beta **(c)** and P53 **(d)**. **C.** FGF19 upregulated genes were involved in WNT signaling **(a)** and ﻿Sonic Hedgehog (SHH) early **(b)** and late**(c)** signaling. **D.** FGF19 up-regulated genes were involved in cell division **(a)** and DNA replication **(b)**.

**Supplementary Figure 3. Overexpression of FGF19 induced cell apoptosis in SK-MES-1 cells.**

The effect of FGF19 overexpression on the cisplatin-induced apoptosis in SK-MES-1 cell line. Left panel, representative flow cytometry graphs; Right panel, histograms of data collected from three independent experiments. ***P < 0.001.

**Supplementary Figure 4. Expression of FGFR4 in patients with LSQ and in vitro evaluation of the effects by inhibition of FGF19 or FGFR4.**

**A.** Protein levels of FGF19 in 20 LSQ samples (T) and its paired Para-Tumor tissues (P) by western blot analysis. **B.** Cells were transfected with FGF19 shRNA. Protein levels of FGF19 and proliferation marker PCNA were determined by western blot. **C. ﻿**Inhibition of FGFR4 by BLU9931 attenuates FGF19-mediated ERK/AKT signaling in HCC15 cells. **D. ﻿**Pan FGFR inhibitor BGJ398 attenuates FGF19-mediated ERK/AKT signaling in H520 cells. **E.** Cells were transfected with either control siRNA, FGF19 siRNA or FGFR4 siRNA. The H520 cells were incubated with the indicated concentrations of Taxol (250 nM) or Cisplatin (500 nM) for 48 h after transfection. Cell viability was determined by CCK-8. **F.﻿** Protein and mRNA expression of EMT markers in the cells transfected with either control siRNA, FGF19 siRNA or FGFR4 siRNA for 48 h in HCC15 cells. **G.** Real-time PCR analysis of stemness-related genes in H520 and HCC15 cells transfected with either control siRNA, FGF19 siRNA or FGFR4 siRNA for 48 h in cells. Data were collected from three independent experiments. *p < 0.05. ** P < 0.01. ***P < 0.001.

**Supplementary Figure 5. ﻿AZD2014 suppresses mTOR pathway induced by FGF19 and﻿ higher expressions of FGF19 and FGFR4, MYC or GLI1 are associated with shorter overall survival in NSCLC.**

**﻿A.**﻿ Western blot analysis shows mTOR pathways inhibited by AZD2014. H520 cells were stimulated with FGF19 (25 ng/mL) for 20 min with or without pretreatment of AZD2014 (1 μM) for 40 min. **B.** Higher total mRNA levels of FGF19 and FGFR4 **(a)**, FGF19 and MYC **(b)**, FGF19 and GLI1 **(c)** are associated with shorter overall survival in NSCLC, evaluated by Kaplan–Meier Plotter (http://www.kmplot.com). Total mRNA level is sum of mRNA levels of FGF19 and selected genes at a 1:1 ratio.

**Supplementary Figure 6. ﻿Effect of mTOR inhibitor AZD2014 in tumor growth of LSQ cells with FGF19 down-regulation in vivo.**

**﻿**HCC15 and H520 cells were transfected with sh-CTRL or sh-FGF19 lentivirus. Cell suspension of HCC15 or H520 (1×10^6^ cells) in a volume of 50 μL was injected subcutaneously into BALB/c nude mice with 15 mg/kg (2 days on/5 days off) of AZD2014 or DMSO by intragastric administration for 3 weeks. **A. ﻿**Representative images **(a)** and quantitative data **(b)** of subcutaneous models in HCC15. IHC analysis of Ki-67 and p-AKT(S473) expression in tumors **(c)** with quantitative data **(d)**. **B.** Images of tumor nodules with luminescent intensities **(a)** and quantitative data **(b)**. IHC analysis of Ki-67 and p-AKT(S473) expression in tumors **(c)** with quantitative data **(d)**.
